# Eunuchs, Circumcision and Leprosy in the East

**Published:** 1857-08

**Authors:** J. Oscar Noyes

**Affiliations:** Late Surgeon in the Ottoman Army


### Eunuchs, Circumcision, and Leprosy in the East.

BY DR. J. OSCAR NOYES, LATE SURGEON IN THE OTTOMAN ARMY.

The Mussulman law distinctly interdicts all mutilations of the human body. Moslems, generally, prefer death to an amputation, or a severe surgical operation of any kind. But, notwithstanding this, the employment of Eunuchs is still retained in the East. Their use by the Asiatic monarchs dates from a remote antiquity. They were numerous in the Eastern Empire before its fall; the Eunuch Narses having been, in fact, one of the best generals of the Greeks. Wherever polygamy has existed these mutilated specimens of humanity have been employed, and probably will be as long as that institution shall have an existence.

While in Constantinople I learned some curious facts relative to Eunuchs, which were confirmed during my visit to Egypt, where they are much more common than in European or Asiatic Turkey, on account of the greater facility in procuring proper subjects. The Turkish grandees obtain their Eunuchs from Egypt, where, in fact, they are exclusively made. The trade is not so active as in former times: as many of the Turks now confine themselves to one wife, there is not so great a demand for these Argus-eyed guardians of Eastern harems. So far as I could learn, about three hundred Eunuchs are annually made in Egypt, some of whom occupy important posts in the Turkish and Egyptian governments. The *Kisler Aga* of the Sultan, for example, is the third man in the Ottoman Empire, having charge not only over the harem of Abdul Medjid, said to contain in all nearly two thousand females, but being also the director of the revenues of the imperial mosques and the incomes derived from Mecca and Medina. From a singular custom of the Ottoman Court, which I am unable to explain, a private harem is kept within the walls of the seraglio for the Kisler Aga, as well as one for the chief of the white Eunuchs.

Syout and Gireh, far up the Nile, are the only places in Egypt where Eunuchs are made for the Egyptian and Turkish markets. The white subjects are Circassian or Georgian boys—the black, Abyssinians or Nubians, from six to nine years of age; the latter being brought by caravans from Sennar and Darfour. The village of *Zawry-el-Dyr*, near Syout, is the great metropolis of the trade in Eunuchs. The perpetrators of this horrid mutilation, to the shame of Christianity be it said, are Christian Copts; and as the subjects of their atrocious cruelty sell from $75 to $200, they drive a very lucrative business for Egypt.

The mutilation—according to the Clot Bey, from whom I have derived much information on this subject—is usually practiced in the Autumn, that season being regarded as most favourable. In most cases the operators do not confine themselves merely to castration, as is generally supposed, but remove with a razor all the exterior organs of generation. Boiling oil is then poured upon the wound, and a short tube inserted into the remaining portion of the urethra, to prevent the closure of the same during the healing process, through which also the boy urinates. A tube is used for the latter purpose throughout life. Powdered hennah is then sprinkled upon the wound, and the sufferer buried for twenty-four hours up to his waist in the sand. Various kinds of unguents are afterwards employed. Three out of every four submitted to the operation perish. Some efforts have of late been made to do away with this barbarous custom, but it will continue in a greater or less degree so long as polygamy shall remain the law of the East. A wealthy Turkish Pacha, wishing to make Abdul Medjid a valuable present, sent him a number of beautiful Circassian boys, who had undergone the operation I have described in Egypt. The Sultan, who is a humane and tender-hearted man, could not repress his indignation at the act, and directed that it should never be attempted again.

The Eunuch can ordinarily be distinguished by his exterior physiognomy. He is usually plethoric, beardless, and has a feminine voice, while a sombre and irascible disposition naturally arises from the sense of degradation which he experiences. From a consciousness of physical inferiority, Eunuchs are usually most bigoted Mussulmans, seeking in the austere practices of religion a substitute for the ordinary pleasures of life. Some of them have a fondness for female society, and there are instances in which they marry. Circumcision is practiced with the Mussulmans, generally about the seventh year.

In Egypt, females are also circumcised by removing a part of the clitoris. The principal object of this is to moderate the *penchant* of the Egyptian females to voluptuousness. It is supposed to have been practiced by the ancient Egyptians, and is not prescribed by the religon of the Moslems.

In the East, young females are valued for their virtue. Effective means are often employed to secure virginity until marriage shall take place. In childhood, the labia are scarified and brought together, when adhesion follows, closing up the vagina, with the exception of a small opening for the urine and menstrual secretion. The jewel, chastity, is in this manner kept safe. After marriage, a slight operation again opens the vagina. By this means an additional value is given to Circassian and Abyssinian slaves. The operation is not performed upon all who are sold, but upon many. It is not practised to any extent with the Nubians, who are less acceptable to the Mussulmans. On the contrary, I have been informed that it is customary for dealers in Nubian girls to deflower them with the finger, with the idea of contributing thereby to their development. I have also heard it stated, with how much truth I am unable to say, that Mussulmans, when long absent on a journey or pilgrimage, sometimes secure the virginity of their daughters, and even the chastity of their wives, by the means above described.

The Egyptian husband must have ocular proof that he has married a virgin; hence, as among the Arabs, the rupture of the hymen is a public rather than a private act. The husband deflowers his wife in the presence of the mothers of both, and other married females belonging to the two families, with the index finger covered with a white muslin handkerchief. Inspired by the most cruel and shameful jealousy, he often employs a brutal violence in the accomplishment of this barbarous act. The handkerchief, covered with blood, is presented to the friends, who felicitate the victim upon her chastity; and the day after the marriage the bloody proof of purity is exhibited publicly by the mother or sister of the bride. If the proof of chastity be wanting, from malformation or previous rupture of the hymen, the female is sent back to her parents, and sometimes even thrown into the Nile. Fortunately, however, this barbarous test of virginity rarely fails; but Egyptian husbands are sometimes duped by artificial means, employed in the absence of a hymen, or even of chastity itself—means well understood by the inmates of the harems.

While in Jerusalem, I devoted some attention to the lepers. Dr. Simms, of the Jews' Hospital, was so kind as to conduct me to their mud kennels in a little enclosure just inside the Sion gate. But few travelers venture into this mephitic retreat, reeking with filth and corruption, for all avoid contact with the lepers. Of these pitiable objects, slunk away in their wretched dens, or lying near the city gates to reach out a trembling hand to the passer-by, there are about thirty in Jerusalem. The disease with which they are afflicted is by no means confined to Palestine, but appears to be more common there than elsewhere; and surely, O reader, one must travel far to behold objects of such intense and corroding misery.

The following description of the leprosy of Palestine, and of the usual course of the disease, is for the most part the result of my own observations upon cases in Jerusalem, some of the suggestions having been borrowed from an excellent article upon the subject, by Dr. Ainslie, published in the Transactions of the Royal Society.

The disease is common in Palestine, and its unfortunate victims are generally to be seen in the towns and cities. It spares no rank or sect, but is more commonly found among the poor than the rich. Aretarus, of Cappadocia, has given us, under the title of *Elephantiasis*, a very perfect description of leprosy. In his account, written in Greek, he makes particular mention of the falling off of the fingers and the joints of the feet. The medical men of his time not unfrequently called the disease *Leonia*, from the circumstance of its distorting the human face so as to give it an appearance somewhat resembling that of the female lion when enraged. Others bestowed upon it the appellation of *Satyriasis*, from the lascivious disposition supposed to be one of its attendant symptoms, when in reality neither food nor rest invigorates the sufferer, and all carnal appetites, instead of being increased, gradually die away.

Leprosy does not often make its appearance before the age of puberty. In cases where in does appear before that age, it represses in a marked degree the growth and development of the body. The stature never becomes full and graceful, but remains shrivelled and meagre—the voice also continuing shrill and nasal. The mind suffers as well as the body. Lepers rarely smile, are drooping and listless, and several of the pitable sufferers in Jerusalem appear to have been actually demented by the disease. The malady commonly manifests itself about the age of twenty-three or twenty-four, and rarely after the age of forty.

The symptoms of *Lepra Arabum*, for that is the scientific name of the disease in question, are as follows:—A dryness and slight roughness of the skin of the hands, feet, arms, and legs, caused by the want of perspiration, are first perceived. The appetite fails, the sleep is disturbed by wild dreams, and the sufferer often wakes up during the night in a fright, with a palpitating heart and a sense of suffocation. In six or eight weeks his color becomes two or three shades darker, and his features slightly tumid. The dryness and roughness of the extremities are followed by numbness and insensibility to pain; the pulse becomes extremely languid, and dark colored spots and purple tubercles usually appear on the wrists and ankles. The latter are not unlike segments of unripe currants in shape, of a shining and oily appearance, but are not attended with any pain.—They increase in size and number, some of them occasionally scaling off to be quickly replaced again, while others generate a small quantity of ichorous matter, which, on drying, occasions a scurvy desquamation. As the leprosy continues to advance, the tubercles extend to the face, and render the person a most unsightly object. At the end of the first year the dryness and rigidity of the skin becomes universal. The numbness extends above the knees, and is so great that the poor sufferer may, inadvertently, burn his hands or feet to the bone without being sensible of it. The surface of the body is wrinkled longitudinally, and where sensibility remains, feels as if stung with nettles.—The countenance undergoes a marked change; the cheeks become bloated and puffy; the muscles of the forehead enlarge, and appear as if pushed downward; and the eyes, in all cases inflamed, rheumy, and made to look rounder than natural by the pressure from the neighboring parts, resemble those of some wild animal. The lobes of the ear are rough, the tongue is foul and in some cases covered with tubercles, the breath is fœtid, the hairs of the head gradually fall off, the nails break and waste away, the fingers and toes appear withered, and the appetite passes entirely away. In this advanced stage the malady will, sometimes, continue for years, but always with progressive misery.

At last when the dyspepsia is most tormenting and the respiration hurried—when the least exertion is sufficient to cause dyaphoresis, although the only parts that perspire are the neck and a little surface around the waist—when the sufferer, already ugly, has become indescribably loathsome—a feverish heat comes on regularly every evening, the eyes assume a dim but brassy appearance, the voice sounds hollow, as if from a tomb, the pulsation can be felt only by pressure over the heart itself, ulcerations take place over the joints, and, to add helplessness to misery, the latter begin one by one to fall off. And thus perishes the poor leper in his filthy den, or at the city gate—perhaps in the vicinity of his synagogue or his mosque.

The leprosy of the Arabians—i. e., the disease with which the thirty lepers of Jerusalem are afflicted—is hereditary, but not contagious. Sometimes, however, it skips over a generation.—There is an example of this kind in Jerusalem, in which the parents are both lepers, while the son, though considerably advanced in years, exhibits no indications of the disease. I examined the leper of Jerusalem, as I would any ordinary cases of disease, without fear of contagion. That the Jews do not consider it contagious is evident from the fact that one of their number, who is afflicted with the malady is permitted to dwell with his friends, and is not cast out among the common lepers.

Women are less subject to leprosy than men, while poor living, want of cleanliness, mendicant misery, and exposure to cold and damp are but too constant attendants of the disease. A fish diet is also found to render every symptom worse. In the East, where the classification of diseases is very imperfect, several kinds of cutaneous affection that are curable, pass for leprosy, but the disease whose symptoms I have described appears to be incurable. Life, however, may be prolonged by careful attention to diet, execise, and cleanliness. Mercury and mineral acids have been employed, but with how much success is not certain. [American Medical Monthly.

